# Cationic Polyene Phospholipids as DNA Carriers for Ocular Gene Therapy

**DOI:** 10.1155/2014/703253

**Published:** 2014-07-24

**Authors:** Susana Machado, Sofia Calado, Diogo Bitoque, Ana Vanessa Oliveira, Christer L. Øpstad, Muhammad Zeeshan, Hans-Richard Sliwka, Vassilia Partali, Michael D. Pungente, Gabriela A. Silva

**Affiliations:** ^1^Center for Biomedicine and Structural Biology (CBME), Institute for Biotechnology and Bioengineering (IBB), University of Algarve, 8005-139 Faro, Portugal; ^2^Doctoral Program in Biomedical Sciences, Department of Biomedical Sciences and Medicine, University of Algarve, 8005-139 Faro, Portugal; ^3^ProRegeM Doctoral Program, Department of Biomedical Sciences and Medicine, University of Algarve, 8005-139 Faro, Portugal; ^4^Department of Chemistry, Norwegian University of Science and Technology, 7491 Trondheim, Norway; ^5^Premedical Unit, Weill Cornell Medical College in Qatar, P.O. Box 24144, Doha, Qatar; ^6^Department of Biomedical Sciences and Medicine, University of Algarve, 8005-139 Faro, Portugal

## Abstract

Recent success in the treatment of congenital blindness demonstrates the potential of ocular gene therapy as a therapeutic approach. The eye is a good target due to its small size, minimal diffusion of therapeutic agent to the systemic circulation, and low immune and inflammatory responses. Currently, most approaches are based on viral vectors, but efforts continue towards the synthesis and evaluation of new nonviral carriers to improve nucleic acid delivery. Our objective is to evaluate the efficiency of novel cationic retinoic and carotenoic glycol phospholipids, designated C20-18, C20-20, and C30-20, to deliver DNA to human retinal pigmented epithelium (RPE) cells. Liposomes were produced by solvent evaporation of ethanolic mixtures of the polyene compounds and coformulated with 1,2-dioleoyl-*sn*-glycero-3-phosphoethanolamine (DOPE) or cholesterol (Chol). Addition of DNA to the liposomes formed lipoplexes, which were characterized for binding, size, biocompatibility, and transgene efficiency. Lipoplex formulations of suitable size and biocompatibility were assayed for DNA delivery, both qualitatively and quantitatively, using RPE cells and a GFP-encoding plasmid. The retinoic lipoplex formulation with DOPE revealed a transfection efficiency comparable to the known lipid references 3*β*-[*N*-(*N*′,*N*′-dimethylaminoethane)-carbamoyl]-cholesterol (DC-Chol) and 1,2-dioleoyl-*sn*-glycero-3-ethylphosphocholine (EPC) and GeneJuice. The results demonstrate that cationic polyene phospholipids have potential as DNA carriers for ocular gene therapy.

## 1. Introduction

Gene therapy is a promising treatment of several pathologies [1] and has been successfully employed to cure eye diseases. The eye possesses several advantages for gene therapy: (1) it is a small organ, requiring only minor amounts of gene carriers; (2) it is a spatially confined and compartmentalized organ; (3) its tissues are readily accessible by microsurgical techniques; (4) it has transparent structures visualized by direct noninvasive techniques; (5) it is separated from systemic circulation by the blood-retinal barrier and therefore is an immune privileged organ; (6) it offers a proximate control (the untreated eye) [2–4]; (7) several eye pathologies are monogenic; and (8) mutations are identified.

Successful gene therapy requires nucleic acid carriers of minimal toxicity allowing long and stable gene expression, such as adenovirus and lentivirus [5]. Nevertheless, the use of viral gene carriers is limited by costly and time-consuming production, restricted cloning capacity, and most important severe immune responses [3, 6]. Nonviral carriers have the potential to overcome these drawbacks, although the efficiency of nonviral carriers still falls behind viral systems [2, 4, 7, 8]. Therefore, there is a need to develop novel synthetic carriers which package and protect the nucleic acid cargo, successfully travel across the cytoplasmic membrane, and ultimately release their nucleic acid load for nuclear uptake [5].

Nonviral carriers are typically cationic polymers or liposomes. Cationic liposomes can easily be produced in large scale [9], can be made tissue specific, have the ability to interact with biological membranes, are well internalized by cells, display low toxicity, and are able to escape the endosome [7].

Cationic lipids interact with negatively charged nucleic acids through a combination of electrostatic and hydrophobic interactions, resulting in lipoplexes of multilamellar structures [7]. The structure and shape of the lipoplex and its lipid/nucleic acid molar ratio are decisive for successful gene delivery [10].

The transfection efficiency and biocompatibility of cationic lipid gene carriers can be altered by modifications of the constituent parts of the lipids, specifically, the lipid backbone (often glycerol), the hydrophilic head group, and the interconnecting linker. Less diversity is reported for the hydrophobic part, which is known to play a significant role in the morphology of the lipoplex [4]. The cationic head group is largely responsible for toxicity and determines the strength of the electrostatic attraction for the negatively charged nucleic acid. Linker groups such as amides, esters, and ethers define the site for lipid cleavage, which is again associated with cytotoxicity [11]. The hydrophobic domain usually consists of one or two saturated or monounsaturated hydrocarbon chains.

**Figure 1:**
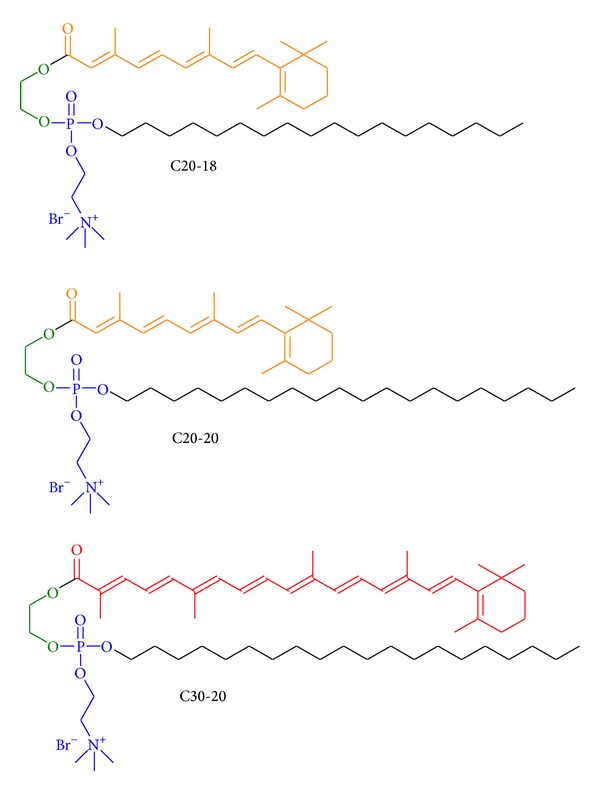
C20-18, C20-20, and C30-20 cationic amphiphilic glycol polyene phospholipids. The color of the polyene chain indicates approximately the visually appearance of the compounds. Glycol backbone green, hydrophilic part blue.

Dietary carotenoids are known to enhance visual performance and to reduce the risk of AMD progression [12]. The glycol lipids, investigated in this report, consist of retinoic (C20:5) and carotenoic (C30:9) polyene chains, combined with a C20:0 and C18:0 chains (Figure 1) [13].

**Figure 2:**
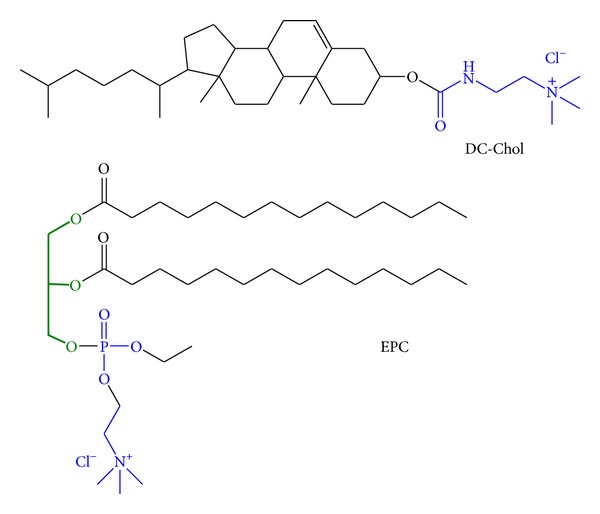
Reference compounds. DC-Chol and cationic glycerophosphospholipid EPC with two C14:0 chains. Glycerol backbone green, hydrophilic parts blue.

Our objective was to evaluate the gene delivery efficiency and cytotoxicity of the cationic polyene glycol lipids against the reference glycerol lipids 3*β*-[*N*-(*N*′,*N*′-dimethylaminoethane)-carbamoyl]-cholesterol (DC-Chol) and 1,2-dioleoyl-*sn*-glycero-3-ethylphosphocholine (EPC; referred to elsewhere as “diC14-EPC” [14]) (Figure 2) in human retinal pigmented epithelium (RPE) cells.

**Figure 3:**
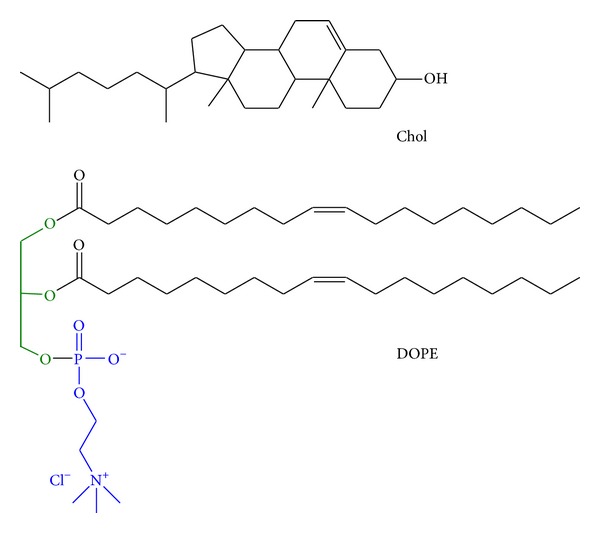
Colipids. Neutral cholesterol (Chol) and zwitterionic glycerophosphospholipid DOPE with two C18:1 chains. Glycerol backbone green, hydrophilic parts blue.

The polyene lipids were formulated with either cholesterol (Chol) or 1,2-dioleoyl-*sn*-glycero-3-phosphoethanolamine (DOPE) (Figure 3), and the impact of these colipids on gene delivery and cellular toxicity was assessed.

## 2. Materials and Methods

### 2.1. General Materials

The ARPE-19 cell line, a human retinal pigment epithelial cell line, was kindly provided by Dr. Franscisco Ambrósio (Center for Neuroscience and Cell Biology, University of Coimbra, Portugal); Dulbecco's modified eagle medium (DMEM) culture medium and Dulbecco's modified eagle medium F12 ham (DMEM F12 Ham) culture medium, fetal bovine serum (FBS), trypsin, glutamine, penicillin-streptomycin solution, dichloromethane, thiazolyl blue tetrazolium bromide (MTT), 0.04 N HCl in 2-propanol constituents, dd-water, tris-acetate-EDTA (TAE) constituents, Avertin anesthesia constituents (tribromoethanol), Triton X-100, Mowiol mounting media constituents, sucrose (saccharose), paraformaldehyde, eosin, acetic acid, dibutyl phthalate xylene (DPX), sodium phosphate, potassium phosphate, and goat serum stock were purchased from Sigma-Aldrich (Portugal). Absolute ethanol was purchased from Merck Millipore (Portugal).

Plasmid DNA containing the reporter gene green fluorescent protein (GFP) was kindly provided by Dr. Jean Bennett (University of Pennsylvania, USA). Agarose was purchased from Invitrogen (Portugal). GreenSafe and GeneJuice were purchased from NZYTech (Portugal). For the phosphate buffered saline (PBS), sodium chloride, potassium chloride, 4′,6-diamidino-2-phenylindole (DAPI), and optimal cutting temperature (OCT) cryostat embedding solution were purchased from VWR (Portugal). Primary antibody Iba1, a microglia marker, was purchased from Wako Pure Chemical Industries (Japan) and secondary antibody Alexa 594 was purchased from Invitrogen (Portugal). Oxalic acid, potassium permanganate (KMnO_4_), and hematoxylin were purchased from Merck (Portugal). Control cationic lipids 3*β*-[*N*-(*N*′,*N*′-dimethylaminoethane)-carbamoyl]-cholesterol (DC-Chol) and 1,2-dimyristoyl-*sn*-glycero-3-ethylphosphocholine (EPC) [14] and the neutral colipids cholesterol (Chol) and 1,2-dioleoyl-*sn*-glycero-3-phosphoethanolamine (DOPE) were obtained from Avanti Polar Lipids (Alabaster, AL, USA).

### 2.2. Cationic Polyene Lipids: C20-18, C20-20, and C30-20

Three cationic polyene lipids, C20-18, C20-20, and C30-20, were synthesized from glycol, retinoic acid (C20:5) or C30-acid (C30:9), and choline precursors as reported elsewhere [13, 15].

### 2.3. Preparation of Lipid Ethanolic Stock Solutions

Ethanolic stock solutions were made separately by first dissolving each cationic lipid and colipid in dichloromethane. The dichloromethane was removed under reduced pressure with a rotary evaporator, and then the residue dissolved in absolute ethanol to a final lipid concentration of 1 mM and subsequently stored at −80°C.

### 2.4. Preparation of Liposome Formulations

Cholesterol and DOPE were employed as neutral colipids [4]. Liposomes of C20-18/Chol, C20-20/Chol, C30-20/Chol, C20-18/DOPE, C20-20/DOPE, and C30-20/DOPE in a lipid/colipid molar ratio of 3 : 2 were generated by mixing ethanolic solutions of cationic lipid and colipid and removing the ethanol under reduced pressure. The resulting thin films were then dissolved in dd-water to a final lipid concentration of 2 mM, and incubated overnight at 4°C. Prior to use, these hydrated liposome stocks were warmed to room temperature and then subjected to ultrasounds for 30 minutes at 60°C. The known cationic lipids DC-Chol and EPC were used as controls.

### 2.5. Preparation of Lipoplex Formulations

The addition of predetermined volumes of negatively charged plasmid DNA to positively charged liposomes resulted in lipoplex formulations of defined nitrogen-to-phosphorus (N/P) molar charge ratios. DNA contained the reporter gene GFP. Lipid concentrations, derived from a 2 mM hydrated stock, were 0.243 mM, 0.162 mM, and 0.081 mM, which correlates with the molar charge ratios of 1.5 : 1, 1 : 1, and 0.5 : 1, respectively. Equal volumes of DNA solution in DMEM or DMEM/F12 Ham culture medium and lipids were mixed and incubated at room temperature for 30 min to allow lipoplex formation.

### 2.6. Evaluation of Lipid-DNA Binding

A gel retardation assay was used to evaluate lipoplex formation, as a neutral or net positive charge associated with the lipid-DNA complex retards migration through agarose gel. Lipoplexes with varying lipid : DNA ratios were prepared as described above in a total volume of 20 *μ*L. Samples were loaded onto a 1% agarose gel impregnated with GreenSafe and electrophoresed at 70 V in tris-acetate-EDTA (TAE) buffer. The DNA bands were observed using an ultraviolet transilluminator.

### 2.7. Physicochemical Characterization of the Lipoplexes

Lipoplexes were prepared in phosphate buffered solution (PBS), for subsequent determination of size and zeta potential by dynamic light scattering (DLS) and laser Doppler anemometry, respectively, using a Zetasizer Nanoseries ZS (Malvern Instruments) by diluting the sample 50x with dd-water.

The size and size distribution of lipoplexes are indicated as displayed by the instrument. Measurement parameters were as follows: a laser wavelength of 633 nm and a scattering angle of 173° (fixed). The samples were loaded into polystyrene cuvettes and three measurements were performed, for which the mean result was recorded. Particles with PdI > 0.7 and *d*_H_ > 2000 nm may be outside of the instrument's reliable range.

### 2.8. Evaluation of the* In Vitro* Cytotoxicity of Lipoplexes

D407 cells were maintained in DMEM culture medium supplemented with 5% FBS, 1% glutamine, and 1% penicillin-streptomycin solution at 37°C with 5% CO_2_. ARPE-19 cells were maintained in DMEM F12 HAM culture medium supplemented with 10% FBS, 1% glutamine, and 1% penicillin/streptomycin at 37°C with 5% CO_2_. For the cytotoxicity assays, cells were seeded at 35,000 cells per well (D407) or 65,000 cells per well (ARPE-19) in 24-well plates in complete medium and incubated for 24 h at 37°C and 5% CO_2_. Cell seeding numbers differ with cell line due to differences in cell doubling rate. After 24 h, cells were washed with PBS and 250 *μ*L lipoplex formulations with various N/P molar charge ratios added to each well. After a 4-hour incubation period, 366 *μ*L of culture medium was added. Cells were incubated for 24, 48, and 72 h. Cell viability was determined by the standard MTT (3-(4,5-dimethylthiazol-2-yl)-2,5-diphenyltetrazolium bromide) assay. Briefly, after the incubation period, 50 *μ*L of a 5 mg/mL MTT in PBS solution was added per well, followed by a 4 h incubation period, after which the culture medium was removed and 500 *μ*L of a 0.04 N HCl in 2-propanol solution was added per well. After dissolution of the formazan crystals, their absorbance was measured at 570 and 630 nm for each well. Cell viability was obtained by measurement of optical densities and normalization against the positive control, consisting of cells grown in standard culture medium.

### 2.9. Evaluation of the* In Vitro* Transfection Efficiency of Lipoplexes

Transfection was determined using cells seeded at 100,000 cells per well (D407) or 250,000 cells per well (ARPE-19) in 6-well plates in complete growth medium and incubated at 37°C with 5% CO_2_ for 24 h. Cell seeding numbers differ with cell line due to differences in cell doubling rate. Immediately prior to transfection, cell monolayers were washed with PBS. The lipoplex formulations at various N/P molar charge ratios were added to each well in culture medium up to 2 mL and incubated for 4 h at 37°C, 5% CO_2_. After this period, the medium containing the lipoplexes was removed, the cells were washed with PBS, and complete growth medium was added and further incubated for 72 h at 37°C, 5% CO_2_. Subsequently, the cells were suspended using trypsin, washed with PBS, and resuspended in 500 *μ*L of PBS for flow cytometry analysis (BD FACSCalibur Flow Cytometer, BD Biosciences). Transfection efficiency was assessed by detection of GFP with results normalized for 10,000 events.

### 2.10. Evaluation of the* In Vivo* Cytotoxicity of Lipoplexes

The* in vivo* compatibility of the C20-20/DOPE/DNA (N/P molar charge ratio 0.5 : 1) was evaluated by intravitreal injection of the lipoplexes dispersed in PBS into C57Bl6 mice. C57BL6 mice (6 months old) were housed at controlled temperature with 12 h light/dark cycle and food and water* ad libitum*. All experimental procedures involving animals were carried out in accordance with the Portuguese and European Union regulations (FELASA) and the Association for Research in Vision and Ophthalmology (ARVO). All experimental injections were performed under anesthesia administered by intraperitoneal injection (250 mg/kg dose). Intravitreal injection was performed under a stereomicroscope (Nikon Stereoscopic Microscope) with a 30-gauge needle. Only one eye was injected; the contralateral eye was left noninjected as control. Fourteen days postinjection, the mice were sacrificed by cervical dislocation and the eyes were enucleated and preserved in PFA 4% at 4°C until further use. The eyes were treated with 30% sucrose solution and embedded in OCT (Optimal Cutting Temperature) embedding solution and stored at −80°C. Cryosections of 10 *μ*m thickness were used for detection of Iba1 (ionized calcium-binding adapter molecule 1, a marker of active microglia used to detect local inflammation) by immunohistochemistry. Cryosections were permeabilized with PBS/0.1% Triton X-100 and washed twice with PBS. A 1 : 1000 dilution of the Iba1 antibody was used, and the samples incubated overnight at 4°C in the dark. Samples were washed three times with PBS/0.1% Triton X-100 and a 1 : 2000 dilution of the secondary antibody Alexa 594 was used for detection and incubated for 1 h, at room temperature, in the dark. Samples were washed three times with PBS/0.1% Triton x-100, mounted, and visualized by fluorescence microscopy (Zeiss Axio Imager Z2 Fluorescence Microscope). Samples were counterstained with DAPI (4′,6-diamidino-2-phenylindole), a marker for nuclear detection. Cryosections were also used for hematoxylin and eosin staining to analyze the retina for its morphology and major signs of inflammation. Sections were washed with distilled water, stained with hematoxylin for 1 min, washed with distilled water, stained with 1% eosin for 5 min, washed with 0.2% acetic acid, and mounted with DPX (dibutyl phthalate xylene). Samples were visualized under bright field (Zeiss Axio Imager Z2 Fluorescence Microscope).

### 2.11. Statistical Analysis

All figures are representative of at least 3 separate experiments. For comparison of multiple sets of data one-way ANOVA, including the Dunnet post-test, was performed. Results are expressed as mean +/− SEM. Statistical analysis was performed using GraphPad Prism 6 Software with *P* < 0.05 considered statistically significant.

## 3. Results

### 3.1. Polyene Lipids Form Stable Lipid-DNA Complexes (Lipoplexes)

The size and surface charge of lipid-DNA complexes composed of the polyene lipids were determined using Dynamic Light Scattering (DLS) (Table 1). The lipoplexes were prepared in PBS, since this was the vehicle used for further experiments. The average size range of lipoplexes composed of reference lipids DC-Chol and EPC was *d*_H_ (hydrodynamic diameter) = 320–2010 nm, while for those composed of C20-18, C20-20, and C30-20 was *d*_H_ = 320–2210 nm. The average size of the lipoplexes did not correlate with increasing N/P ratio, with the exception of EPC/DOPE and C20-18/Chol (denoted by ∗), and also did not correlate with formulation of the same lipid with different colipids, Chol, and DOPE, with the exception of EPC and C30-20 lipoplexes (denoted by †). DLS is a technique that depends on the scattering of light with the particles and polydispersity may “hide” real populations due to the presence of large particles. Table 1 shows the mode, which is the most frequent size in the samples. If samples are monodisperse, *d*_H_ values are representative of the sample and will be similar to the mode. However if the sample is polydisperse, *d*_H_ values represent less accurately the sample and its value is apart from the mode [16].

The surface charge values (as measured by zeta potential, ZP, Table 1) indicate stable particles. The lipoplexes of the reference lipids revealed ZP = −54 ± 2– − 69 ± 3 mV, whereas the polyene lipoplexes revealed a broader range with ZP = −30 ± 2– − 66 ± 1 mV. The ZP ranges did not correlate with particle size, as the reference lipids were found to have a narrow ZP range yet generally broader particle distribution compared with the polyene lipoplexes. Significant size changes were not associated with significant changes in ZP. Furthermore, ZP values are not correlated with increasing N/P ratio, except for C30-20/Chol.

**Figure 4:**
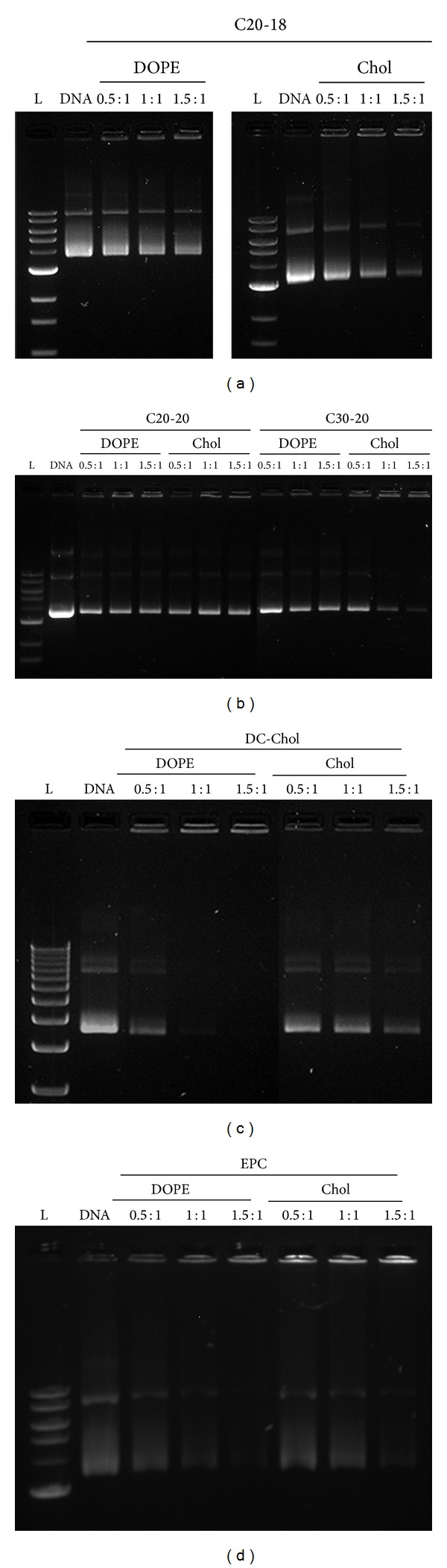
Gel retardation assays with lipoplexes C20-18 (a), C20-20 and C30-20 (b), DC-Chol (c), and EPC (d) with DOPE and Chol as colipids. Molar charge ratios N/P used were 0.5 : 1, 1 : 1, and 1.5 : 1. Retention of DNA increases with increasing molar charge ratios.

The cationic liposomes were combined with a GFP-coded plasmid DNA to evaluate relative gene transfer efficiency to RPE cells. The DNA binding efficiency of C20-18, C20-20, C30-20, DC-Chol, and EPC-containing lipoplexes at various N/P molar charge ratios was assessed using a gel retardation assay. The negatively charged plasmid DNA, when interacting with sufficient cationic lipids, forms a neutral or net positive complex that will not migrate through the agarose gel. As seen in Figure 4, all formulations revealed partial or total DNA retention, demonstrating cationic lipid/DNA lipoplex formation. An increase in N/P molar charge ratio resulted in an increase in DNA retention. C20-18/Chol and C30-20/Chol formulations revealed a higher degree of complexation with DNA, as revealed by a diminished DNA migration band, when compared with C20-18, C20-20, and C30-20 formulations (Figures 4(a) and 4(b)). The reference lipids DC-Chol and EPC showed a similar trend, with higher DNA retention with increasing N/P molar ratios (Figures 4(c) and 4(d)). Our plasmid binding results are in accordance with previous outcomes [13, 15], where C20-20 and C30-20 lipoplexes formulated with DNA also revealed partial retention in a gel retardation assay at low N/P charge ratios.

### 3.2. Toxicity of the Lipoplexes Is Concentration Dependent in RPE Cells

**Figure 5:**
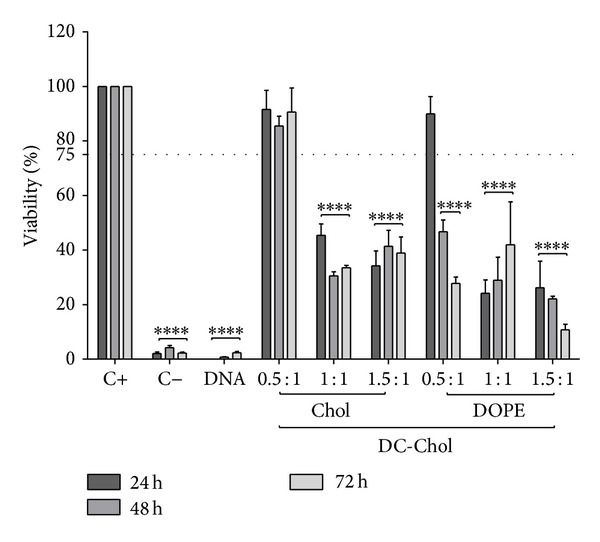
DC-Chol lipoplex cytotoxicity. ARPE-19 cell viability for DC-Chol lipoplexes with either Chol or DOPE as colipid at various N/P ratios incubated up to 72 h. Horizontal line at 75% viability represent the threshold according to the ISO standard for* in vitro* testing of biological devices. C+ represents untreated cells and C− represents cells treated to induce cell death. Statistical significance (∗) of 95% (*P* < 0.05).

**Figure 6:**
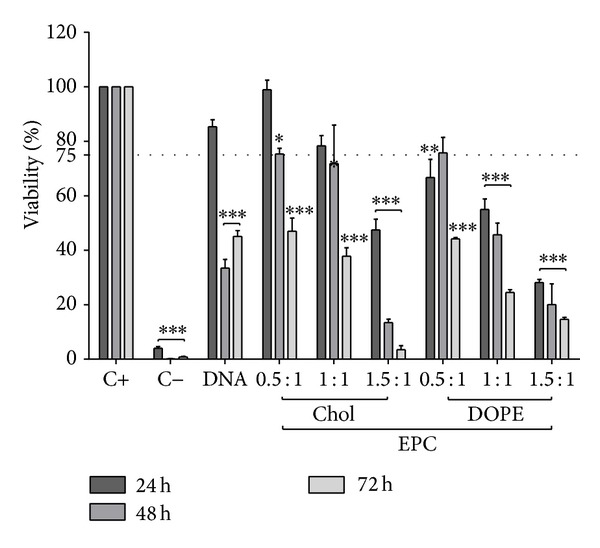
EPC lipoplex cytotoxicity. ARPE-19 cell viability for EPC lipoplexes with either Chol or DOPE as colipids at various N/P molar charge ratios incubated up to 72 h. Horizontal line at 75% viability represents the threshold according to the ISO standard for* in vitro* testing of biological devices. C+ represents untreated cells and C− represents cells treated to induce cell death. Statistical significance (∗) of 95% (*P* < 0.05).

**Figure 7:**
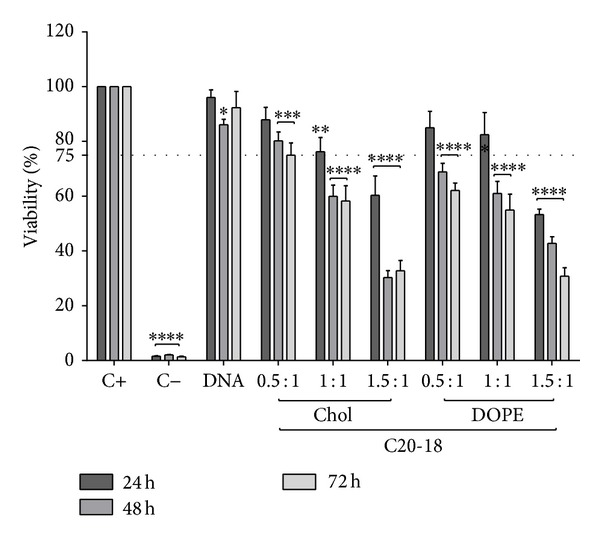
C20-18 lipoplex cytotoxicity. ARPE-19 cell viability for C20-18 lipoplexes with either Chol or DOPE as colipid at various N/P ratios incubated up to 72 h. Horizontal line at 75% viability represents the threshold according to the ISO standard for* in vitro* testing of biological devices. C+ represents untreated cells and C− represents cells treated to induce cell death. Statistical significance (∗) of 95% (*P* < 0.05).

**Figure 8:**
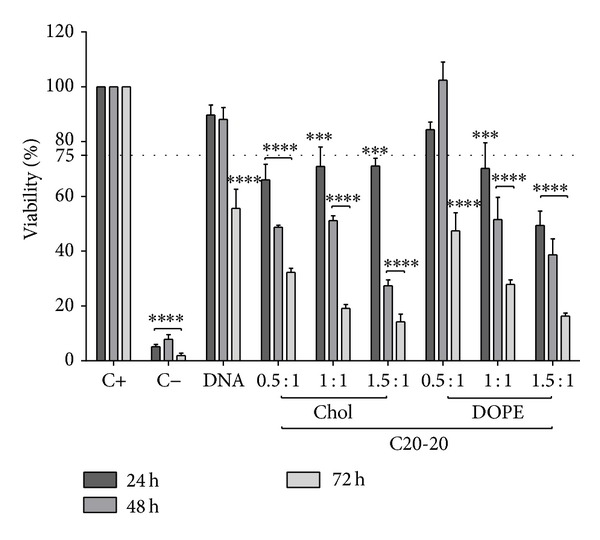
C20-20 lipoplex cytotoxicity. ARPE-19 cell viability for C20-20 lipoplexes with either Chol or DOPE as colipid at various N/P ratios incubated up to 72 h. Horizontal line at 75% viability represents the threshold according to the ISO standard for* in vitro* testing of biological devices. C+ represents untreated cells and C− represents cells treated to induce cell death. Statistical significance (∗) of 95% (*P* < 0.05).

**Figure 9:**
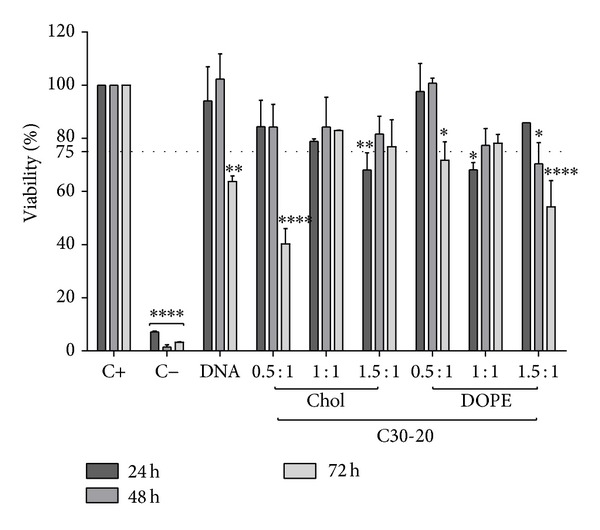
C30-20 lipoplex cytotoxicity. ARPE-19 cell viability for C30-20 lipoplexes with either Chol or DOPE as colipid at various N/P ratios incubated up to 72 h. Horizontal line at 75% viability represents the threshold according to the ISO standard for* in vitro* testing of biological devices. C+ represents untreated cells and C− represents cells treated to induce cell death. Statistical significance (∗) of 95% (*P* < 0.05).

ARPE-19 cells were cultured in the presence of C20-18, C20-20, C30-20, DC-Chol, and EPC lipoplexes at varying N/P molar charge ratios, and cell viability was assessed after 24, 48, and 72 h (Figures 5, 6, 7, 8, and 9). A decrease in cell viability below 75% was defined as the cytotoxicity threshold, according to the ISO standard for biological evaluation of medical devices [17].

Toxicity was concentration dependent (increasing with increased N/P molar charge ratio) and time dependent (duration of the incubation period) as previously described [18]. Lipoplex C20-20 was found to be more cytotoxic than C20-18 and C30-20. The reference lipids exhibit toxicity comparable with C20-20.

Based on the cytotoxicity results, the 0.5 : 1 N/P molar charge ratio was subsequently used in transfection assays and* in vivo* assays for all formulations due to lower cytotoxicity.

### 3.3. Toxicity of C20-20/DOPE Lipoplex under* In Vivo* Conditions

**Figure 10:**
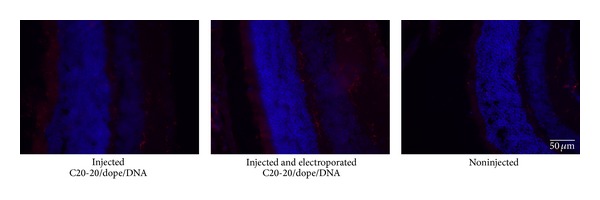
Immunohistochemistry detection of Iba1 in mouse retinas injected with C20-20/DOPE lipoplexes at the N/P molar charge ratio 0.5 : 1. Magnification: 100x and scale bar: 50 *μ*m.

To evaluate whether the level of toxicity observed* in vitro* was similar for* in vivo*, C20-20/DOPE lipoplexes prepared in PBS at a N/P molar charge ratio of 0.5 : 1 were administered by intravitreal injection to the eyes of C57BL6 mice. After 14 days, mice were sacrificed and the eyes were enucleated and used for Iba1 detection by immunohistochemistry or stained with hematoxylin and eosin for assessment of retinal integrity and inflammation. Iba1 is a calcium-binding protein characteristic of microglia that is expressed when the microglia is reactive, that is, in situations where inflammation occurs [19]. Our results showed that retinas of mice injected with C20-20/DOPE lipoplexes at the N/P molar charge ratio 0.5 : 1 (Figure 10) revealed microglia in the nonreactive state, with short cellular ramifications, indicating the absence of inflammation in the retina. The control, which is the contralateral noninjected eye, also showed similar microglia morphology in its nonreactive state. These results confirm that C20-20 is not toxic when administered* in vivo* in the retina of C57Bl6 mice.

**Figure 11:**
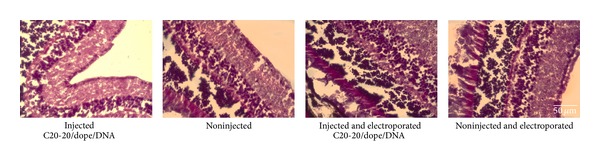
Hematoxylin and eosin staining of mouse retinas injected with C20-20/DOPE lipoplexes at the N/P molar charge ratio 0.5 : 1, showing the maintenance of the integrity of the retinal layered structure. Magnification: 100x and scale bar: 50 *μ*m.

Administration of drugs by intravitreal injection can result in morphological disruption of the retinal structure, caused by the injection procedure and tissue inflammation. Hematoxylin and eosin staining (Figure 11) showed no morphological disruption of the retina in the* in vivo* study.

### 3.4. Transfection Efficiency of C20-18, C20-20, and C30-20 Lipoplexes in RPE Cells

**Figure 12:**
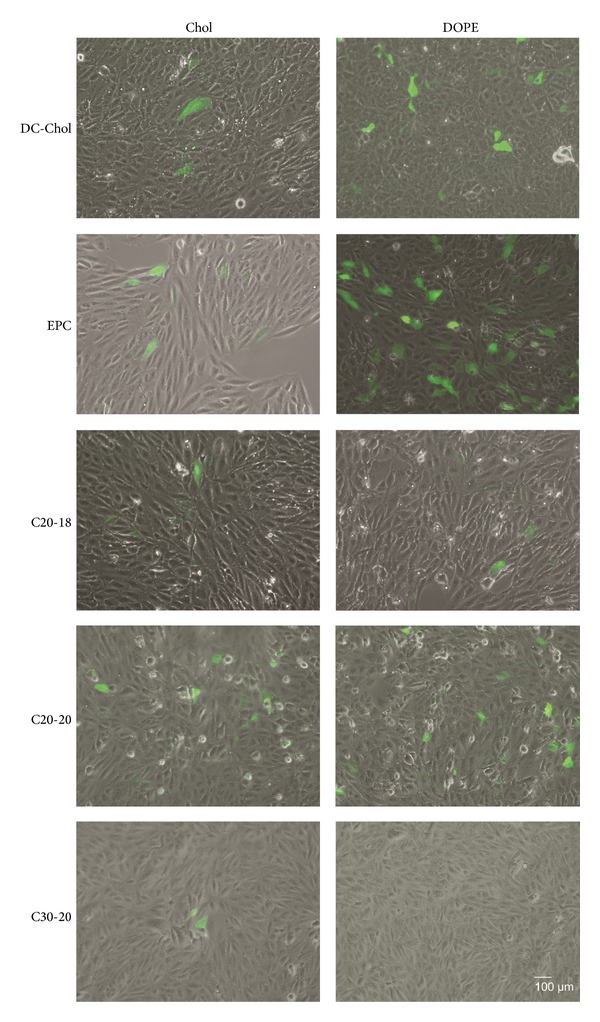
Qualitative assessment of lipoplex transfection. ARPE-19 cells were incubated with lipoplexes containing lipids C20-18, C20-20, or C30-20 against reference lipids DC-Chol and EPC with either Chol or DOPE as colipid at N/P molar ratio 0.5 : 1 for 4 h and GFP-expressing cells evaluated by fluorescence microscopy after 72 h. Magnification: 100x and scale bar: 100 *μ*m.

**Figure 13:**
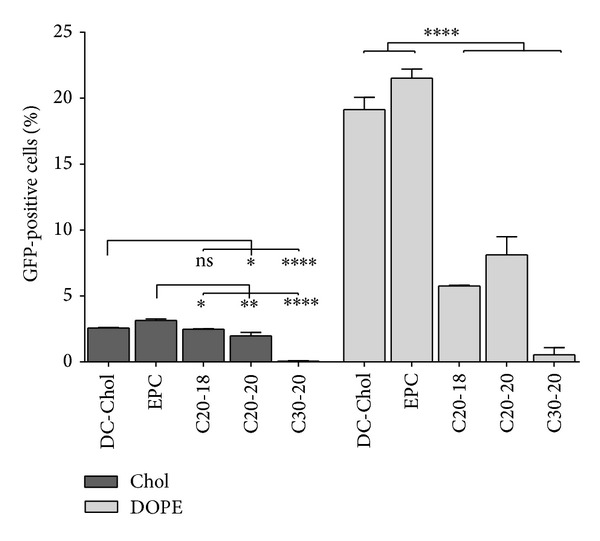
The transfection efficiency of lipoplexes. ARPE-19 cells were incubated with lipoplexes containing lipids C20-18, C20-20, or C30-20 against reference lipids DC-Chol and EPC with either Chol or DOPE as colipid at N/P molar ratio 0.5 : 1 for 4 h and transfection efficiencies, measured by GFP expression, determined by flow cytometry after 72 h. Statistical significance (∗) of 95% (*P* < 0.05).

We investigated the* in vitro* transfection efficiency of the C20-18, C20-20, and C30-20 lipoplexes in retinal cells using a plasmid coding for GFP and assessing its expression qualitatively by fluorescence microscopy and quantitatively by flow cytometry. ARPE-19 cells were transfected with C20-18, C20-20, and C30-20 lipoplexes with either Chol or DOPE as colipid at a N/P molar ratio of 0.5 : 1; transfection was evaluated by GFP expression after 72 h (Figures 12 and 13 and Supplementary Figure S2 available online at http://dx.doi.org/10.1155/2014/703253). For polyene and reference lipids alike, all DOPE formulated lipoplexes, with the exception of C30-20, revealed greater transfection efficiency than those formulations with Chol as colipid (Figure 13). Of the polyene lipids studied, the C20-20/DOPE formulation revealed the greatest transfection efficiency, with an average of 8%, followed by C20-18/DOPE with 6% (Figure 13). Upon comparison of the polyene lipoplexes with DC-Chol and EPC lipoplexes, C20-18 and C20-20 lipoplexes revealed greater competitive transfection efficiencies when Chol was used as colipid (Figures 12 and 13).

The transfection efficiency of C20-20 and C30-20 lipoplexes at N/P molar charge ratio of 0.5 : 1 containing either Chol or DOPE as colipid was also assessed in D407 cells against the positive control, GeneJuice (supplementary Figure S1). As was found with the ARPE-19 cell line, the C20-20/DOPE formulation outperformed the corresponding Chol formulation in the D407 cell line (supplementary Figure S1). In addition, C20-20/DOPE revealed a level of transfection competitive to the control GeneJuice, whereas the C30-20 lipid revealed only a low level of transfection with Chol as colipid.

## 4. Discussion

Cationic polyene lipids were evaluated for their potential to carry DNA into retinal cells. Transfection efficiency was correlated with molecular structure, lipoplex size, zeta potential, biocompatibility, and the presence of colipids. DOPE and cholesterol increase DNA delivery and thus were employed as colipids. DOPE, often referred to as a “fusogenic lipid,” could alter the packing of the hydrophobic chains within the lipoplex [10] by promoting a nonlamellar structure, thus destabilizing the endosomal membrane and releasing DNA [7] upon membrane fusion between the lipoplex and the endosome [8]. The addition of plasmid DNA to the self-aggregated liposomes formed lipoplexes, which were characterized for their physical properties and capacity of DNA complexation. All liposomes showed some degree of complexation with plasmid DNA; however, complete retention of the lipoplexes in the gel retardation assay was not always observed. With the exception of C20-20, the polyene lipids complexed DNA more effectively when cholesterol was used as colipid, while EPC and DC-Chol showed more complete binding with formulations that contained DOPE (Figure 2). It was expected that increasing the N/P molar ratio for those lipoplex formulations with incomplete gel retardation would result in complete DNA retention [20] at the expense of cell viability.

The analysis of the physical properties of the lipoplexes, summarized in Table 1, shows that some EPC and DC-Chol formulations gave rise to large lipoplex sizes and high polydispersity index (PdI). This is most likely due to aggregation and fusion of liposomes during lipoplex formation [21]. Despite this observation, the sizes of the lipoplexes are in the range of those generally considered adequate for* in vitro* cell internalization, although a correlation between size, surface charge, and transfection efficiency is still debatable [10].

Almofti et al. demonstrated for the cationic lipid DC-6-14 that lipoplex size increased lipoplex transfection [22]. With the exception of DC-Chol, all DOPE formulations revealed better transfection efficiencies with larger lipoplex compared to Chol formulations. This can be explained by the multiple endocytic mechanisms existing in the cells for internalization of lipoplexes of different sizes [7].

Zeta potential reflects the surface charge and lipoplex stability: ZP > ±30 mV indicates stable particles, and ZP < ±10 mV suggests unstable particles with a tendency to aggregation [10].

Our lipoplexes presented negative zeta potential values between −30 ± 2 and −66 ± 1 mV. Although a positively charged lipoplex interacts more favorably with the negative charged biological membranes, it is not a necessary condition for transfection [9] and, as shown in Figures 12 and 13, C20-20/DOPE and C20-18/DOPE lipoplexes were able to transfect retinal cells.

Negative ZP values most likely derive from DNA bound to the outer shell of the lipoplexes and from the phosphate groups associated with the solvent (PBS) that remains close to the surface of the lipoplexes.

Successful gene carriers do not only transfect efficiently, but they also need to be biocompatible with the organism and target tissue.* In vitro* testing showed that the lipoplexes have increased* in vitro* toxicity with increasing N/P molar ratio and incubation period, in line with reference lipids DC-Chol, EPC, and other cationic lipids [18, 23]. Toxicity is lipoplex specific and tissue specific, dose dependent and route dependent. Lipoplex formulations can be modified to reduce toxicity or to promote transfection efficiency; one example is PEGylation, which can control lipoplex size and increase circulation time* in vivo* [7]. In the retina, toxicity and inflammation can be evaluated by morphological analysis of the retina and by immunohistochemistry detection of inflammatory markers, such as the microglia. The microglia consists of macrophages and monocytes and is a normal constituent in the retina, mainly distributed in the nerve fiber layer and ganglion cell layer [24]. In its nonreactive state, ramified microglia can be observed in the tissue. However, when tissue is damaged, microglial cells migrate to all retinal layers, retract cell processes (ramifications), and increase cell size [25]. Our results show* in vivo* compatibility of C20-20 with the retinal tissue, as shown by the absence of morphological alterations in the retina and by nonreactive microglia.

The efficiency of* in vitro* gene delivery was evaluated with a plasmid coding for the GFP reporter gene, through detection of GFP-positive cells. C20-20 and the C20-18 formulations showed greater transfection efficiency relative to C30-20 when either DOPE or Chol was used as colipid. All lipid-DOPE formulations showed enhanced transfection efficiency when compared with lipid-Chol formulations. DOPE probably facilitates lipoplex phase transitions from lamellar to inverted hexagonal packing, which allows an effective fusion of the lipoplex and the cellular endocytic membrane [26, 27]. It has previously been demonstrated that lipoplexes of EPC, C20-20, and C30-20, with either DOPE or Chol, gave rise to lamellar morphologies as shown by small-angle X-ray scattering [12]. However, only those lamellar lipoplexes containing DOPE are expected to invert to hexagonal packing in the endosome.

Although transfection in ARPE-19 cells was higher for the DC-Chol/DOPE and EPC/DOPE lipoplexes, transfection efficiencies of the C20-18/DOPE and C20-20/DOPE lipoplexes are noteworthy when compared to other investigations reporting considerably lower efficiencies [28].

In an attempt to further compare the relative transfection efficiency of C20-20 and C30-20, we tested the same formulations in D407 cells (supplementary Figure S1), another cell line from the retina, but with a faster mitotic rate than ARPE-19. We observed with these cells a greater transfection efficiency than with ARPE-19 cells, which is most likely due to the higher mitotic rate, since during mitosis the nuclear membrane is disaggregated and therefore nuclear penetration of nucleic acids is facilitated.

Remarkably, C20-20 displayed greater transgene efficiency than C30-20 in both cell lines tested. The retinoic lipids C20-18 and C20-20 may therefor enter the nucleus via a possible retinoid receptor [29]. Further studies are required to investigate a possible retinoid receptor mediated nuclear uptake mechanism.

C20-18/Chol and C20-20/Chol performed comparably to references DC-Chol and EPC. C20-20/DOPE lipoplexes delivered DNA* in vitro* to RPE cells and were biocompatible during* in vivo* applications. These results encourage us to optimize and characterize this process.

## 5. Conclusion

Cationic liposomes formulated from polyene lipids C20-18, C20-20, and C30-20 and a neutral colipid were combined with a GFP-coded plasmid DNA for studies in retina cells. Gel electrophoresis was used to confirm the formation of lipoplexes in all formulations containing either DOPE or Chol as colipid. The diameters of lipoplexes were relatively large and variable; however, this did not appear to hinder transfection, since for a given cationic lipid larger lipoplexes gave rise to greater transfection (Table 1 and Figure 13). Toxicity associated with the polyene lipids under* in vitro* conditions was, as anticipated, concentration dependent and increased with increasing N/P ratio. Toxicity was also found to increase with increasing incubation period, in line with the formulations for reference lipids DC-Chol and EPC.

C20-20 was found to have greater* in vitro* cytotoxicity than C20-18 or C30-20. Nonetheless, C20-20 showed no evidence of inflammation in the mouse retina.* In vitro* transfection efficiencies of 6% and 8% were achieved with ARPE-19 cells with C20-18/DOPE and C20-20/DOPE, respectively. Furthermore, DNA delivery using C20-20/DOPE outperformed C30-20 in D407 retinal cells and revealed a level of transfection competitive to reference GeneJuice.

Overall, C20-20/DOPE gave promising results for ocular gene therapy. Future work is directed to optimize formulations for* in vivo* ocular gene delivery.

## Supplementary Material

S1: Transfection efficiency of novel cationic polyene lipids in another retinal cell line, D407. Comparison with Gene Juice, a commercial reagent, shows a similar trend.
S2: Flow cytometry scatter plots for cell transfection with the novel cationic polyene lipids.

## Figures and Tables

**Table 1:** Size and surface charge (as measured by zeta potential, ZP) of C20-18, C20-20, C30-20, DC-Chol, and EPC lipoplexes in PBS at varying N/P molar charge ratios with either Chol or DOPE as colipids.

Lipid	Colipid	*N*/*P* ratio	*d*_ H_ (nm)	Mode (nm)	Pdl	ZP (mV)
**DC-Chol**		**0.5 : 1**	1180	477	0.718	−55 ± 5.61
	**Chol**	**1 : 1**	1480	455	0.840^†^	−55 ± 5.19
		**1.5 : 1**	1220	516	0.611	−65 ± 3.11
		**0.5 : 1**	900	441	0.664	−63 ± 0.65
	**DOPE**	**1 : 1**	1310	600	0.424	−69 ± 2.76
		**1.5 : 1**	1290	506	0.768∗	−65 ± 1.06

**EPC**		**0.5 : 1**	510^†^	488	0.220^†^	−54 ± 1.65
	**Chol**	**1 : 1**	360	380	0.058	−57 ± 1.01
		**1.5 : 1**	320^†^	322^†^	0.234^†^	−57 ± 0.85
		**0.5 : 1**	1410	528	0.750	−57 ± 1.22
	**DOPE**	**1 : 1**	930	625	0.366	−58 ± 1.96
		**1.5 : 1**	2010∗	656	0.862	−54 ± 1.98

**C20-18**		**0.5 : 1**	320	256	0.322	−41 ± 10.81
	**Chol**	**1 : 1**	390	361	0.285	−51 ± 4.57
		**1.5 : 1**	1523∗	490	0.702∗	−43 ± 8.11
		**0.5 : 1**	550	487	0.425	−53 ± 6.73
	**DOPE**	**1 : 1**	910	406	0.624	−38 ± 7.77
		**1.5 : 1**	1270	453	0.596	−36 ± 1.74

**C20-20**		**0.5 : 1**	560	548	0.130	−53 ± 0.27
	**Chol**	**1 : 1**	570	537	0.157	−59 ± 1.03
		**1.5 : 1**	660	537	0.257	−61 ± 0.37
		**0.5 : 1**	640	465	0.304	−53 ± 1.43
	**DOPE**	**1 : 1**	970	500	0.155	−41 ± 0.88
		**1.5 : 1**	750	594	0.147	−62 ± 0.50

**C30-20**		**0.5 : 1**	500^†^	355^∗†^	0.353	−36 ± 1.43*
	**Chol**	**1 : 1**	820	763	0.178	−60 ± 1.86^†^
		**1.5 : 1**	710	697	0.260	−66 ± 0.58^†^
		**0.5 : 1**	1500	937∗	0.144	−30 ± 1.71
	**DOPE**	**1 : 1**	970	605	0.419	−40 ± 1.43
		**1.5 : 1**	1140	768	0.220	−40 ± 0.12

^†^Comparison between Chol and DOPE formulations of one lipid with the same molar charge ratio; *Comparison between ratios (0.5 : 1 versus 1 : 1 or 1 : 1 versus 1.5 : 1) of the same lipid formulation.
