# Supplementary figures and images for: Cationic Polyene Phospholipids as DNA Carriers for Ocular Gene Therapy
Source: Biomed Res Int. 2014 Jul 24;2014:703253. doi: 10.1155/2014/703253 (PMC4131563; doi:10.1155/2014/703253)

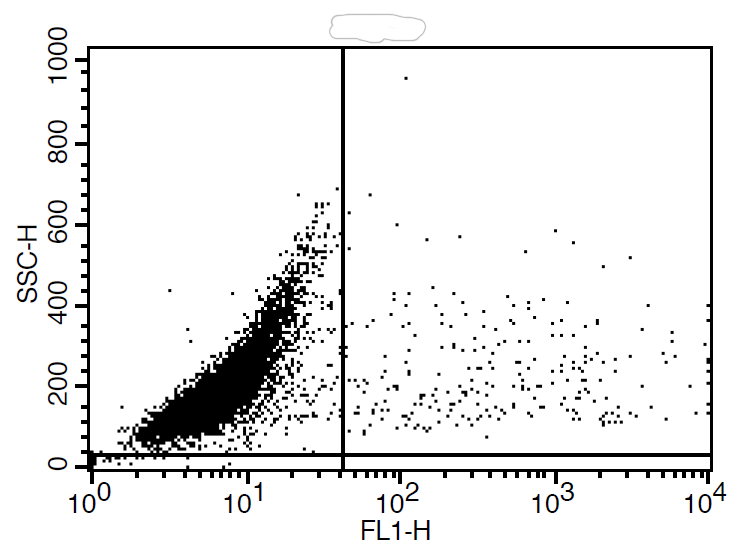

Supplement: Supplementary file 1 — S1: Transfection efficiency of novel cationic polyene lipids in another retinal cell line, D407. Comparison with Gene Juice, a commercial reagent, shows a similar trend. S2: Flow cytometry scatter plots for cell transfection with the novel cationic polyene lipids. [file 703253.f1.zip › C20-18 1.tif]

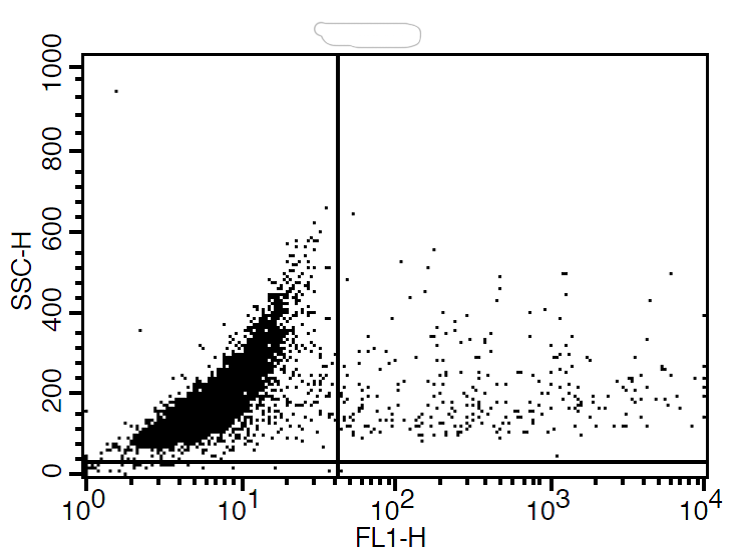

Supplement: Supplementary file 1 — S1: Transfection efficiency of novel cationic polyene lipids in another retinal cell line, D407. Comparison with Gene Juice, a commercial reagent, shows a similar trend. S2: Flow cytometry scatter plots for cell transfection with the novel cationic polyene lipids. [file 703253.f1.zip › C20-18 2.tif]

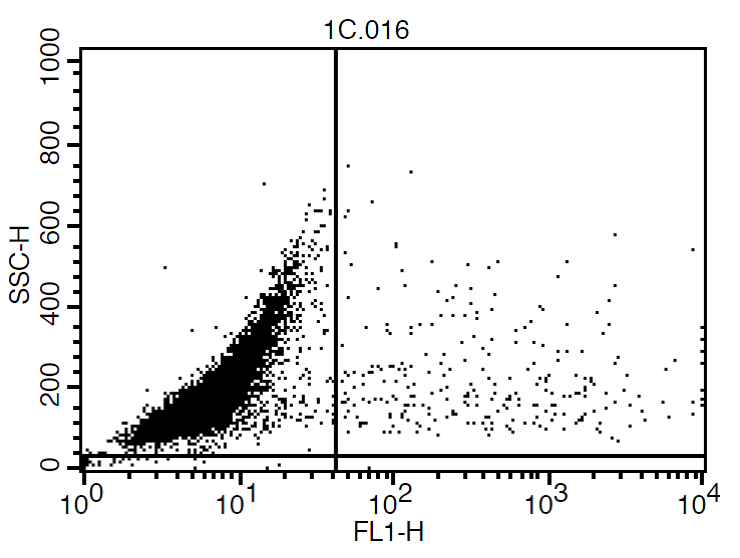

Supplement: Supplementary file 1 — S1: Transfection efficiency of novel cationic polyene lipids in another retinal cell line, D407. Comparison with Gene Juice, a commercial reagent, shows a similar trend. S2: Flow cytometry scatter plots for cell transfection with the novel cationic polyene lipids. [file 703253.f1.zip › C20-18 3.tif]

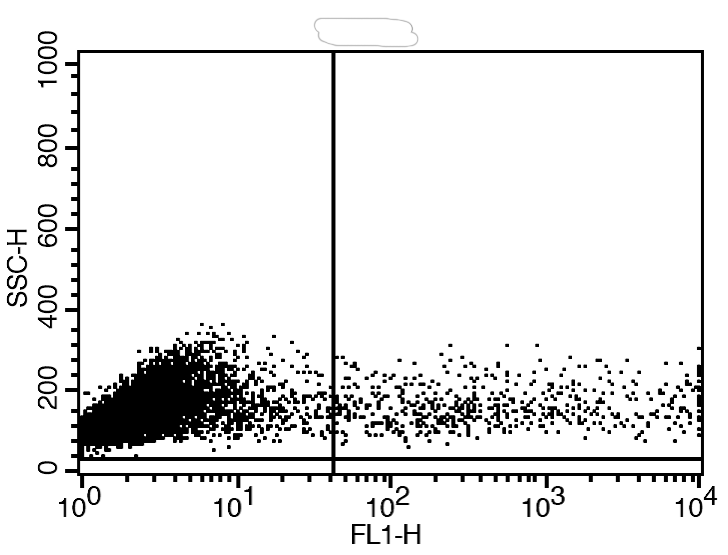

Supplement: Supplementary file 1 — S1: Transfection efficiency of novel cationic polyene lipids in another retinal cell line, D407. Comparison with Gene Juice, a commercial reagent, shows a similar trend. S2: Flow cytometry scatter plots for cell transfection with the novel cationic polyene lipids. [file 703253.f1.zip › C20-18 4.tif]

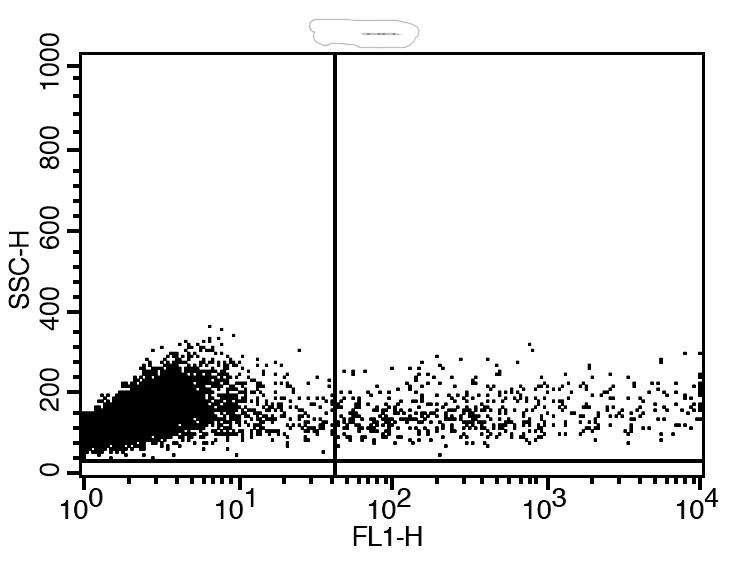

Supplement: Supplementary file 1 — S1: Transfection efficiency of novel cationic polyene lipids in another retinal cell line, D407. Comparison with Gene Juice, a commercial reagent, shows a similar trend. S2: Flow cytometry scatter plots for cell transfection with the novel cationic polyene lipids. [file 703253.f1.zip › C20-18 5.tif]

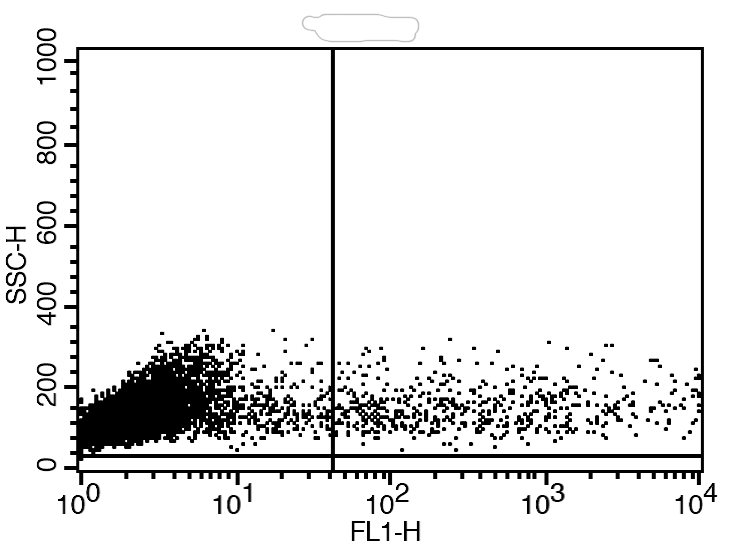

Supplement: Supplementary file 1 — S1: Transfection efficiency of novel cationic polyene lipids in another retinal cell line, D407. Comparison with Gene Juice, a commercial reagent, shows a similar trend. S2: Flow cytometry scatter plots for cell transfection with the novel cationic polyene lipids. [file 703253.f1.zip › C20-18 6.tif]

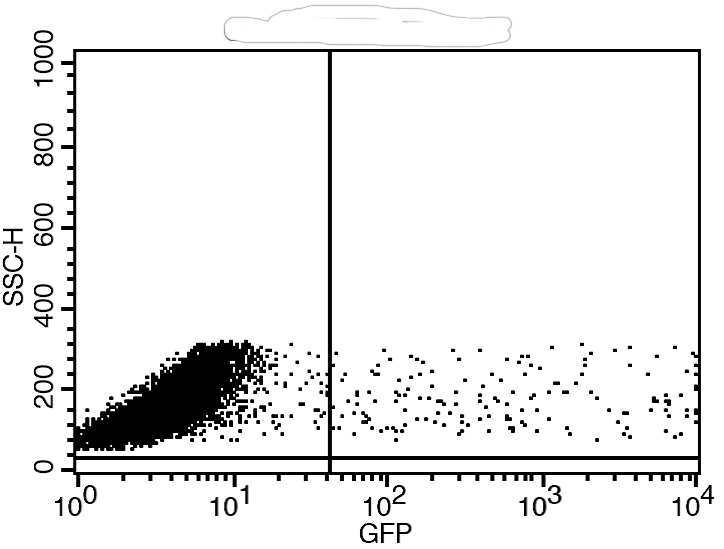

Supplement: Supplementary file 1 — S1: Transfection efficiency of novel cationic polyene lipids in another retinal cell line, D407. Comparison with Gene Juice, a commercial reagent, shows a similar trend. S2: Flow cytometry scatter plots for cell transfection with the novel cationic polyene lipids. [file 703253.f1.zip › C20-20 1.tif]

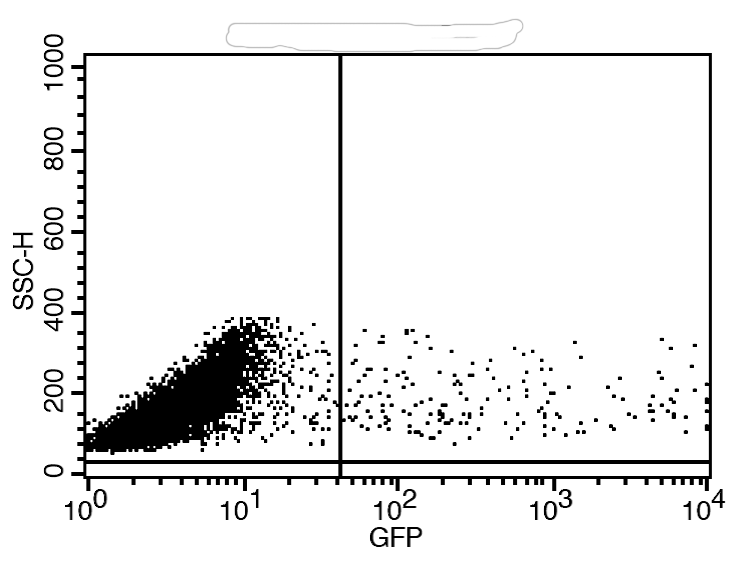

Supplement: Supplementary file 1 — S1: Transfection efficiency of novel cationic polyene lipids in another retinal cell line, D407. Comparison with Gene Juice, a commercial reagent, shows a similar trend. S2: Flow cytometry scatter plots for cell transfection with the novel cationic polyene lipids. [file 703253.f1.zip › C20-20 2.tif]

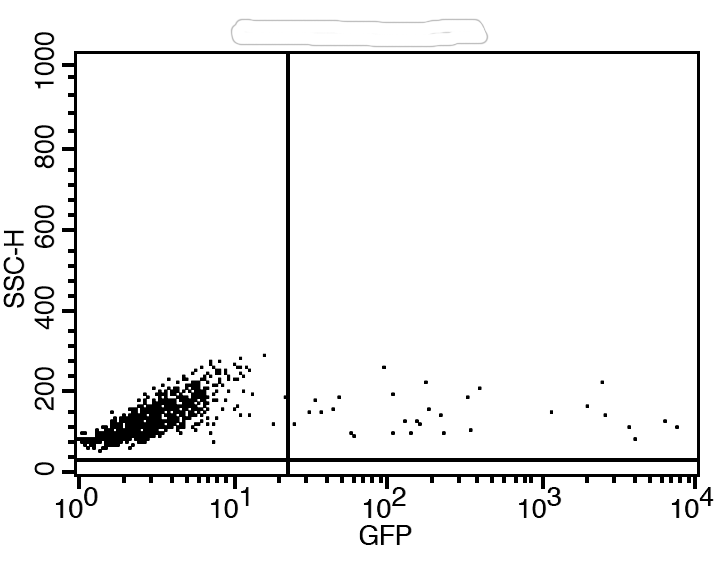

Supplement: Supplementary file 1 — S1: Transfection efficiency of novel cationic polyene lipids in another retinal cell line, D407. Comparison with Gene Juice, a commercial reagent, shows a similar trend. S2: Flow cytometry scatter plots for cell transfection with the novel cationic polyene lipids. [file 703253.f1.zip › C20-20 3.tif]

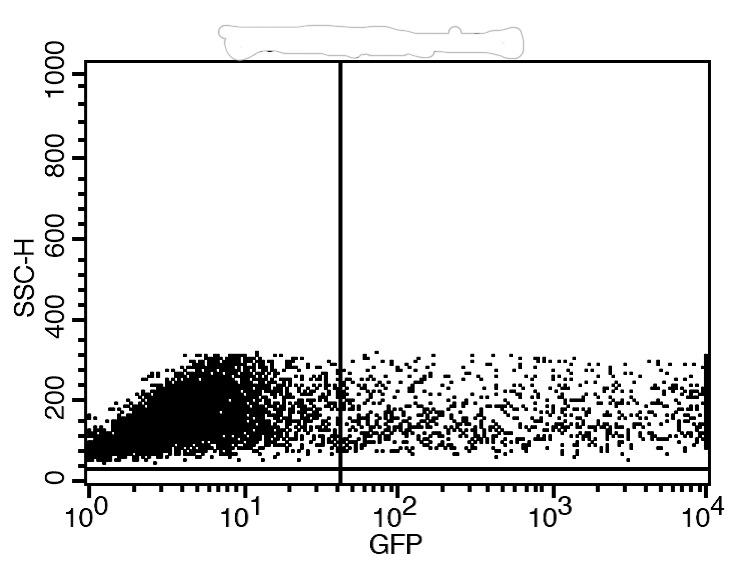

Supplement: Supplementary file 1 — S1: Transfection efficiency of novel cationic polyene lipids in another retinal cell line, D407. Comparison with Gene Juice, a commercial reagent, shows a similar trend. S2: Flow cytometry scatter plots for cell transfection with the novel cationic polyene lipids. [file 703253.f1.zip › C20-20 4.tif]

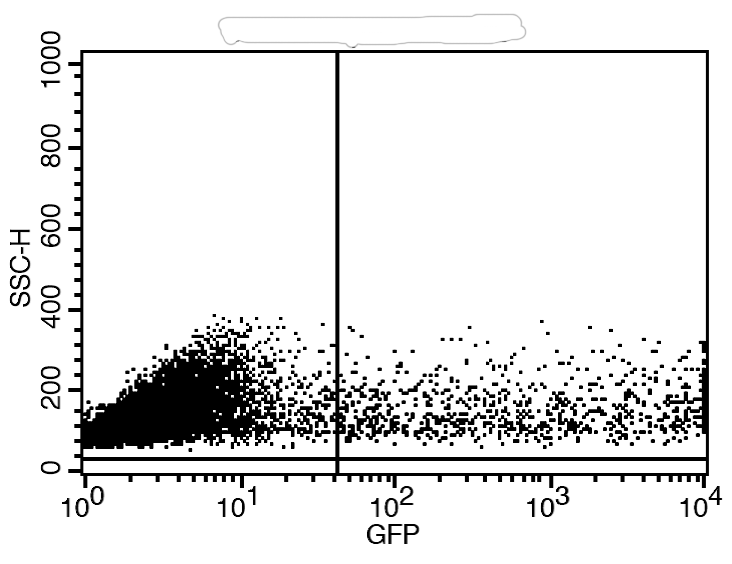

Supplement: Supplementary file 1 — S1: Transfection efficiency of novel cationic polyene lipids in another retinal cell line, D407. Comparison with Gene Juice, a commercial reagent, shows a similar trend. S2: Flow cytometry scatter plots for cell transfection with the novel cationic polyene lipids. [file 703253.f1.zip › C20-20 5.tif]

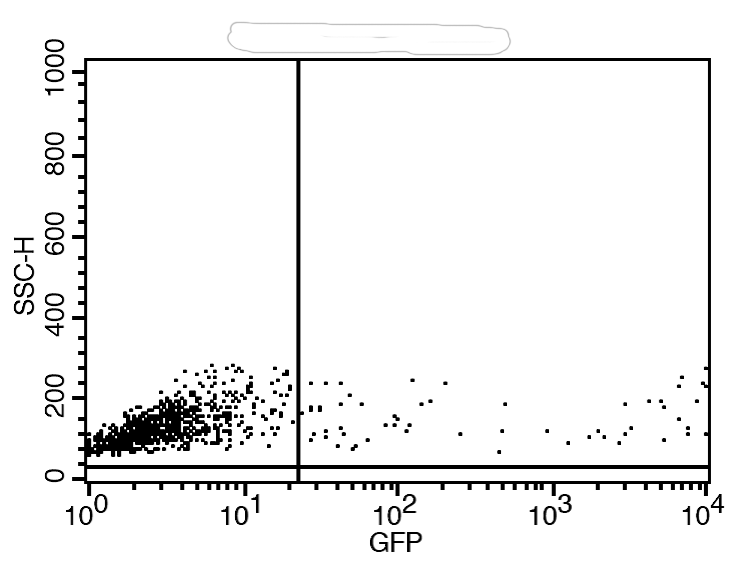

Supplement: Supplementary file 1 — S1: Transfection efficiency of novel cationic polyene lipids in another retinal cell line, D407. Comparison with Gene Juice, a commercial reagent, shows a similar trend. S2: Flow cytometry scatter plots for cell transfection with the novel cationic polyene lipids. [file 703253.f1.zip › C20-20 6.tif]

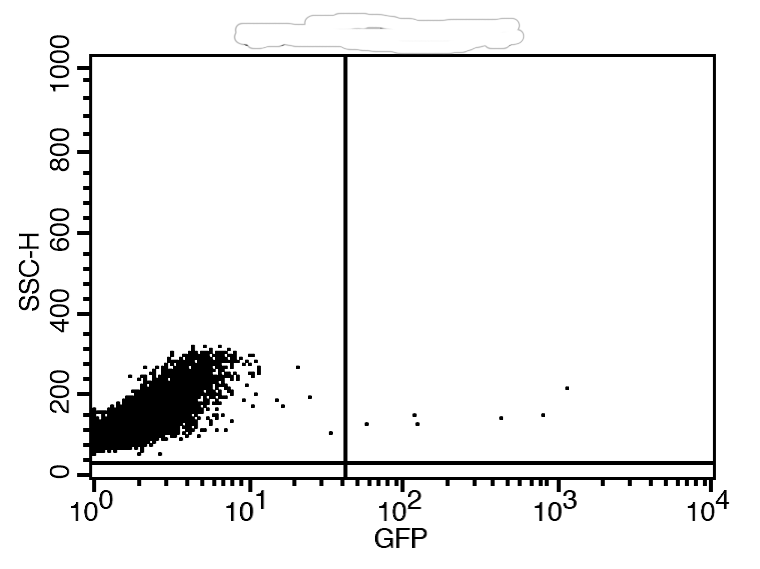

Supplement: Supplementary file 1 — S1: Transfection efficiency of novel cationic polyene lipids in another retinal cell line, D407. Comparison with Gene Juice, a commercial reagent, shows a similar trend. S2: Flow cytometry scatter plots for cell transfection with the novel cationic polyene lipids. [file 703253.f1.zip › C30-20 1.tif]

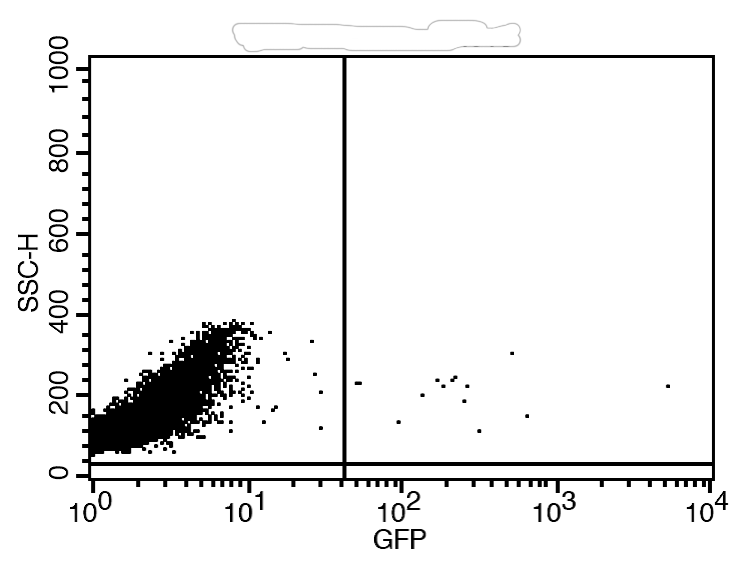

Supplement: Supplementary file 1 — S1: Transfection efficiency of novel cationic polyene lipids in another retinal cell line, D407. Comparison with Gene Juice, a commercial reagent, shows a similar trend. S2: Flow cytometry scatter plots for cell transfection with the novel cationic polyene lipids. [file 703253.f1.zip › C30-20 2.tif]

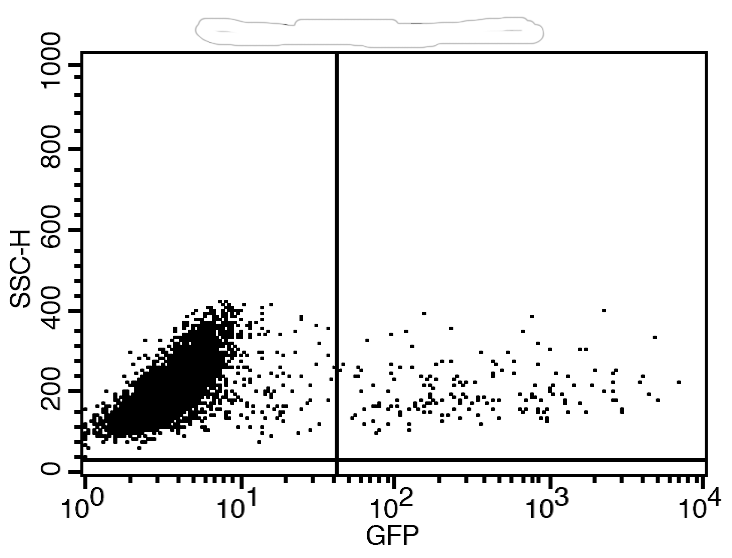

Supplement: Supplementary file 1 — S1: Transfection efficiency of novel cationic polyene lipids in another retinal cell line, D407. Comparison with Gene Juice, a commercial reagent, shows a similar trend. S2: Flow cytometry scatter plots for cell transfection with the novel cationic polyene lipids. [file 703253.f1.zip › C30-20 3.tif]

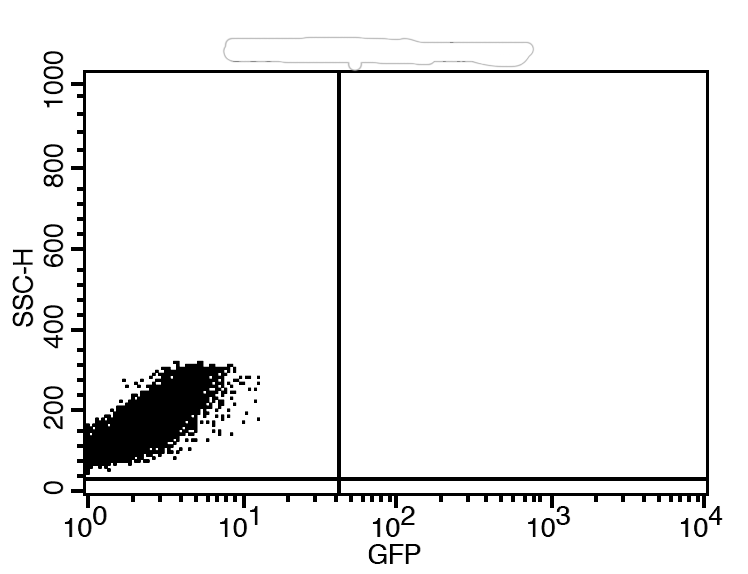

Supplement: Supplementary file 1 — S1: Transfection efficiency of novel cationic polyene lipids in another retinal cell line, D407. Comparison with Gene Juice, a commercial reagent, shows a similar trend. S2: Flow cytometry scatter plots for cell transfection with the novel cationic polyene lipids. [file 703253.f1.zip › C30-20 4.tif]

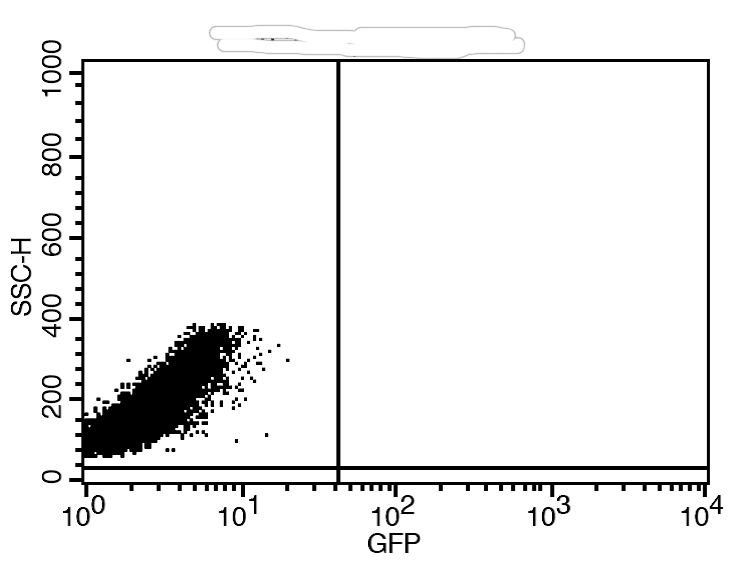

Supplement: Supplementary file 1 — S1: Transfection efficiency of novel cationic polyene lipids in another retinal cell line, D407. Comparison with Gene Juice, a commercial reagent, shows a similar trend. S2: Flow cytometry scatter plots for cell transfection with the novel cationic polyene lipids. [file 703253.f1.zip › C30-20 5.tif]

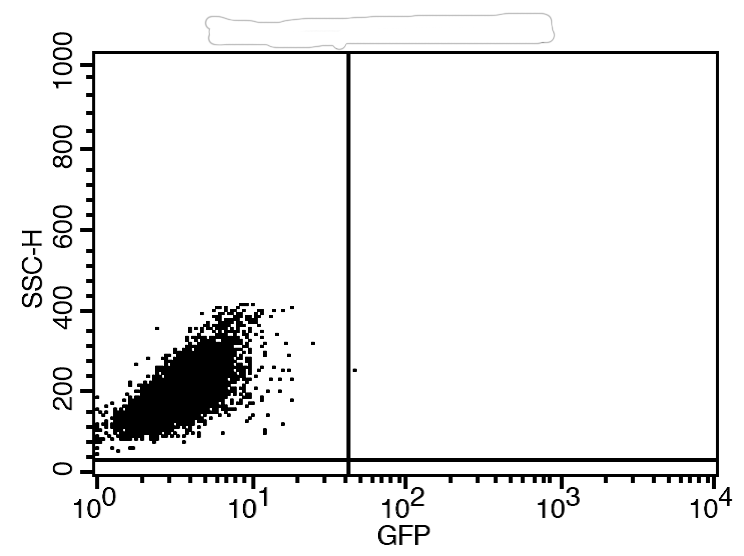

Supplement: Supplementary file 1 — S1: Transfection efficiency of novel cationic polyene lipids in another retinal cell line, D407. Comparison with Gene Juice, a commercial reagent, shows a similar trend. S2: Flow cytometry scatter plots for cell transfection with the novel cationic polyene lipids. [file 703253.f1.zip › C30-20 6.tif]

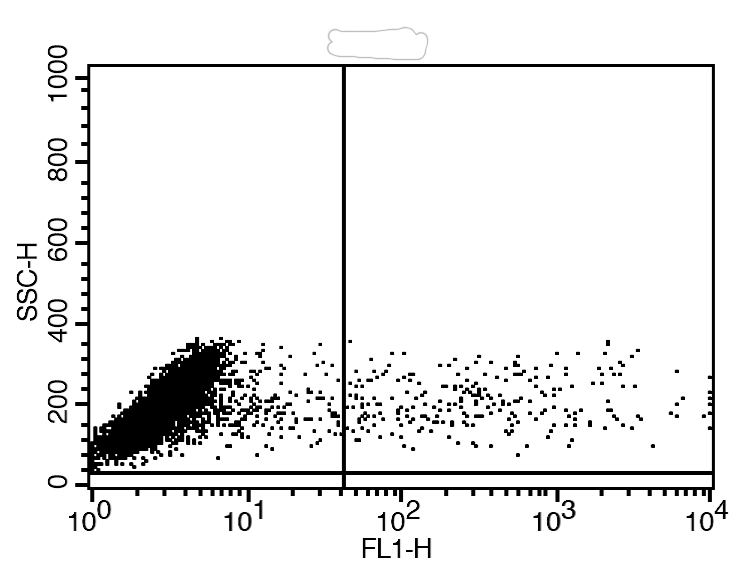

Supplement: Supplementary file 1 — S1: Transfection efficiency of novel cationic polyene lipids in another retinal cell line, D407. Comparison with Gene Juice, a commercial reagent, shows a similar trend. S2: Flow cytometry scatter plots for cell transfection with the novel cationic polyene lipids. [file 703253.f1.zip › DC-Chol 1.tif]

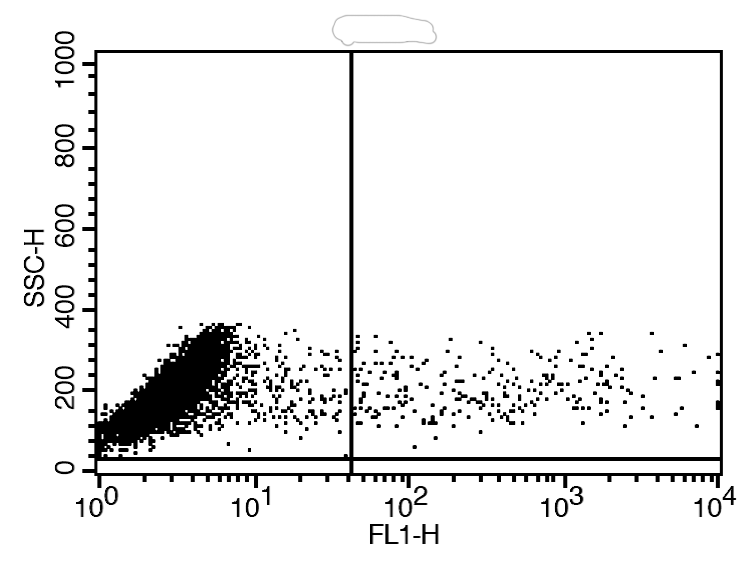

Supplement: Supplementary file 1 — S1: Transfection efficiency of novel cationic polyene lipids in another retinal cell line, D407. Comparison with Gene Juice, a commercial reagent, shows a similar trend. S2: Flow cytometry scatter plots for cell transfection with the novel cationic polyene lipids. [file 703253.f1.zip › DC-Chol 2.tif]

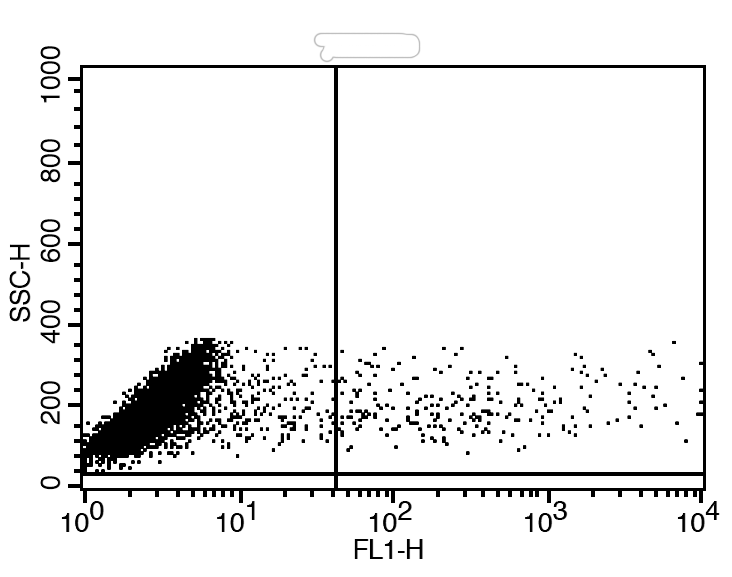

Supplement: Supplementary file 1 — S1: Transfection efficiency of novel cationic polyene lipids in another retinal cell line, D407. Comparison with Gene Juice, a commercial reagent, shows a similar trend. S2: Flow cytometry scatter plots for cell transfection with the novel cationic polyene lipids. [file 703253.f1.zip › DC-Chol 3.tif]

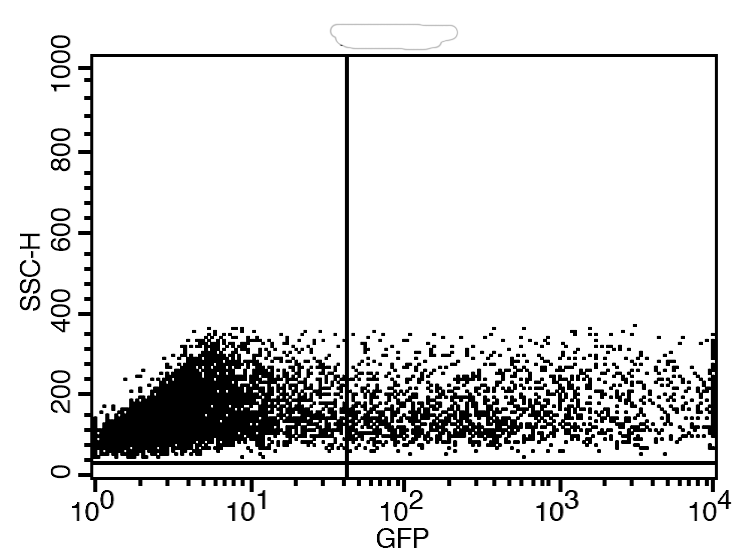

Supplement: Supplementary file 1 — S1: Transfection efficiency of novel cationic polyene lipids in another retinal cell line, D407. Comparison with Gene Juice, a commercial reagent, shows a similar trend. S2: Flow cytometry scatter plots for cell transfection with the novel cationic polyene lipids. [file 703253.f1.zip › DC-Chol 4.tif]

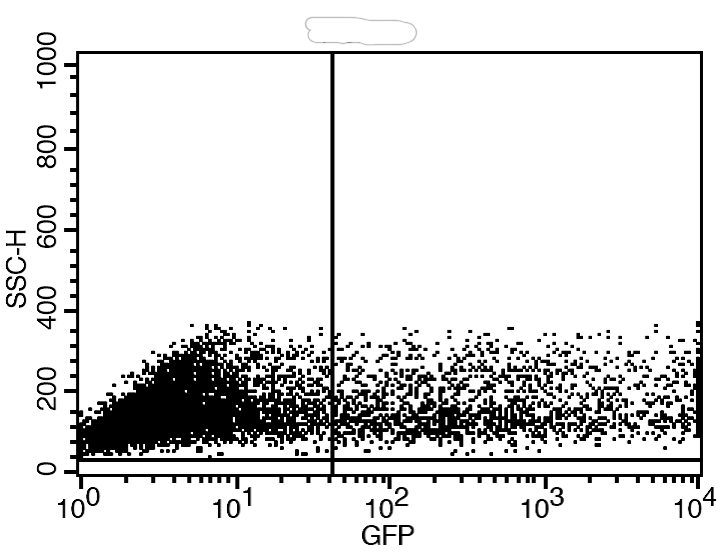

Supplement: Supplementary file 1 — S1: Transfection efficiency of novel cationic polyene lipids in another retinal cell line, D407. Comparison with Gene Juice, a commercial reagent, shows a similar trend. S2: Flow cytometry scatter plots for cell transfection with the novel cationic polyene lipids. [file 703253.f1.zip › DC-Chol 5.tif]

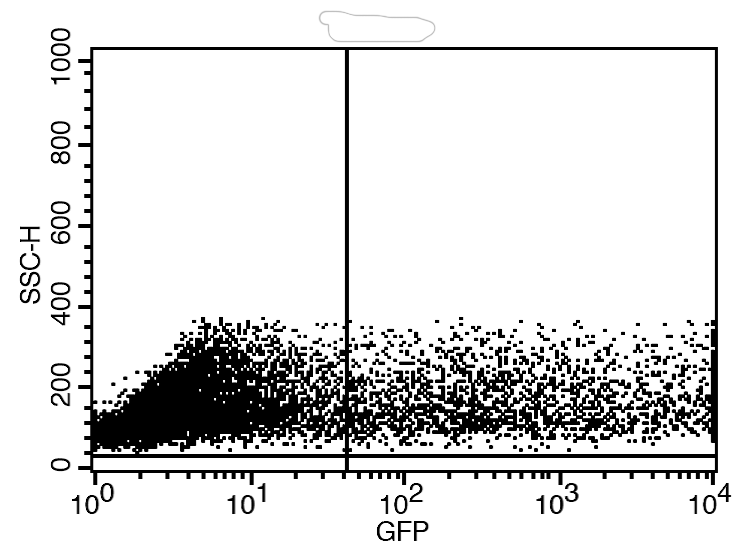

Supplement: Supplementary file 1 — S1: Transfection efficiency of novel cationic polyene lipids in another retinal cell line, D407. Comparison with Gene Juice, a commercial reagent, shows a similar trend. S2: Flow cytometry scatter plots for cell transfection with the novel cationic polyene lipids. [file 703253.f1.zip › DC-Chol 6.tif]

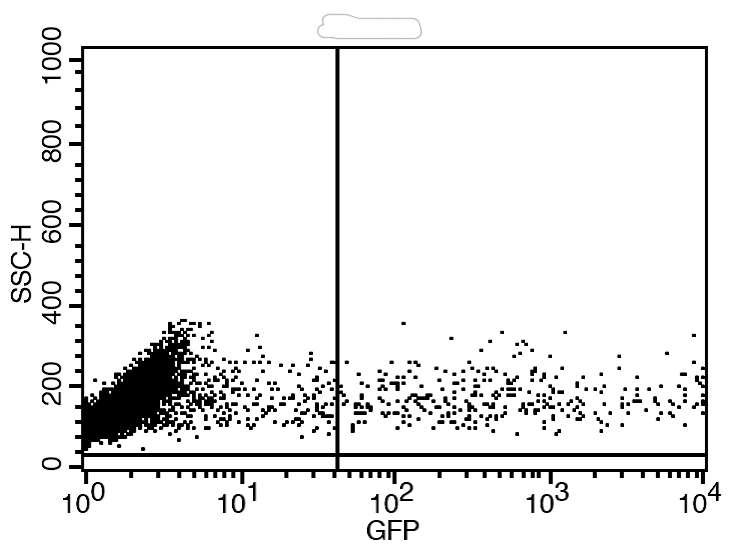

Supplement: Supplementary file 1 — S1: Transfection efficiency of novel cationic polyene lipids in another retinal cell line, D407. Comparison with Gene Juice, a commercial reagent, shows a similar trend. S2: Flow cytometry scatter plots for cell transfection with the novel cationic polyene lipids. [file 703253.f1.zip › EPC 1.tif]

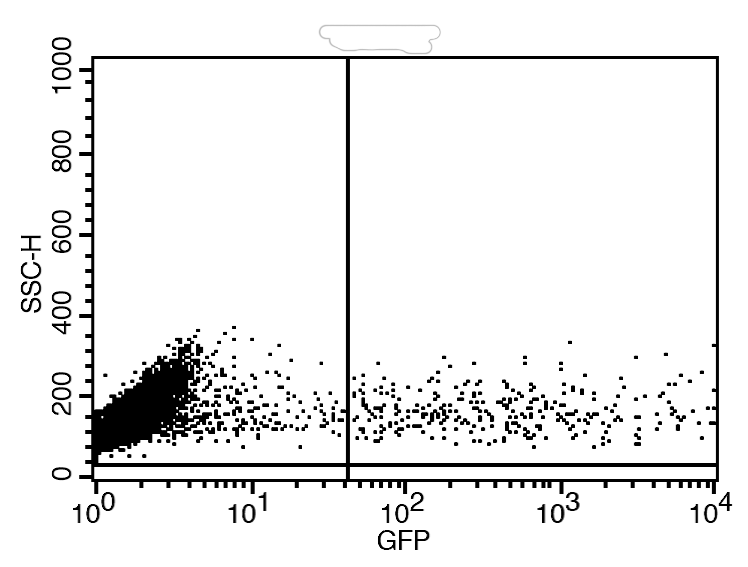

Supplement: Supplementary file 1 — S1: Transfection efficiency of novel cationic polyene lipids in another retinal cell line, D407. Comparison with Gene Juice, a commercial reagent, shows a similar trend. S2: Flow cytometry scatter plots for cell transfection with the novel cationic polyene lipids. [file 703253.f1.zip › EPC 2.tif]

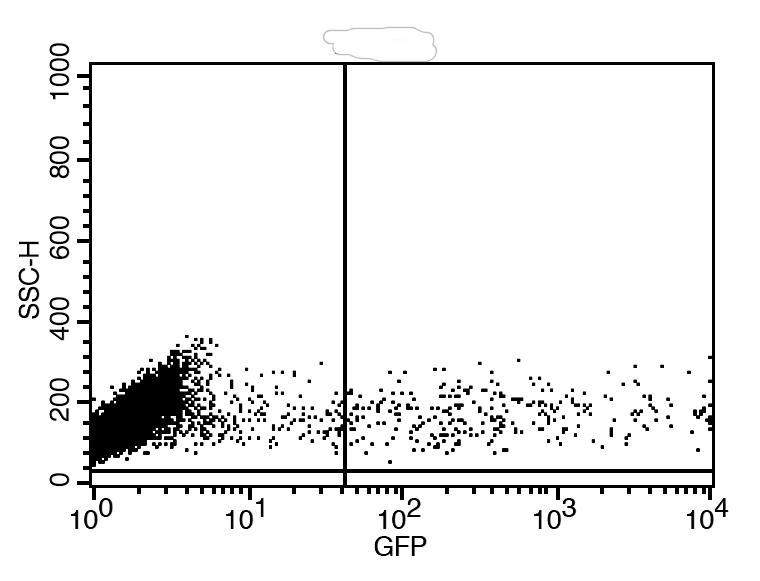

Supplement: Supplementary file 1 — S1: Transfection efficiency of novel cationic polyene lipids in another retinal cell line, D407. Comparison with Gene Juice, a commercial reagent, shows a similar trend. S2: Flow cytometry scatter plots for cell transfection with the novel cationic polyene lipids. [file 703253.f1.zip › EPC 3.tif]

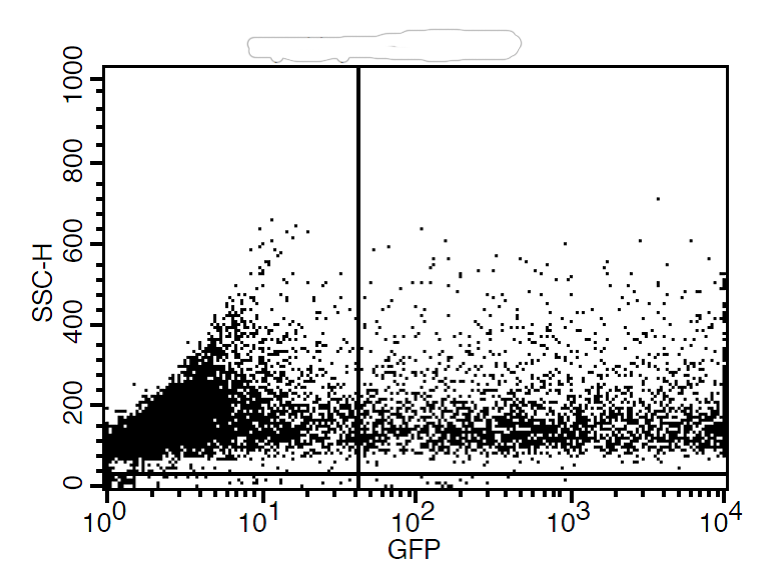

Supplement: Supplementary file 1 — S1: Transfection efficiency of novel cationic polyene lipids in another retinal cell line, D407. Comparison with Gene Juice, a commercial reagent, shows a similar trend. S2: Flow cytometry scatter plots for cell transfection with the novel cationic polyene lipids. [file 703253.f1.zip › EPC 4.tif]

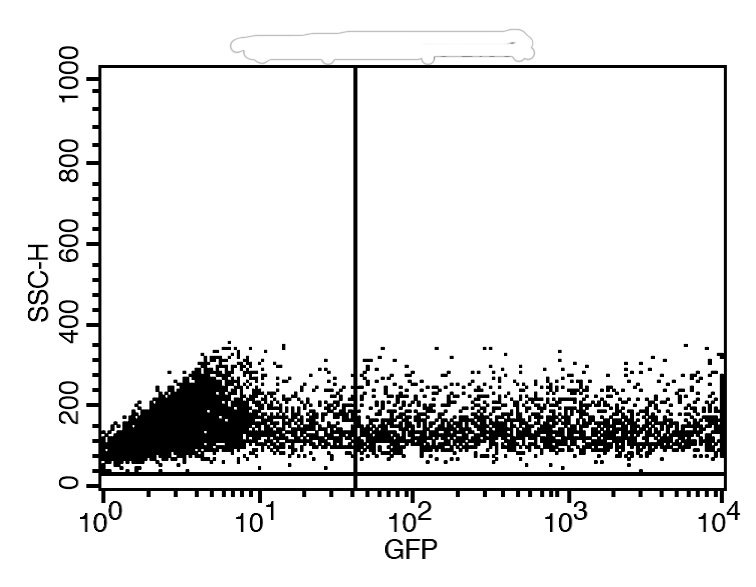

Supplement: Supplementary file 1 — S1: Transfection efficiency of novel cationic polyene lipids in another retinal cell line, D407. Comparison with Gene Juice, a commercial reagent, shows a similar trend. S2: Flow cytometry scatter plots for cell transfection with the novel cationic polyene lipids. [file 703253.f1.zip › EPC 5.tif]

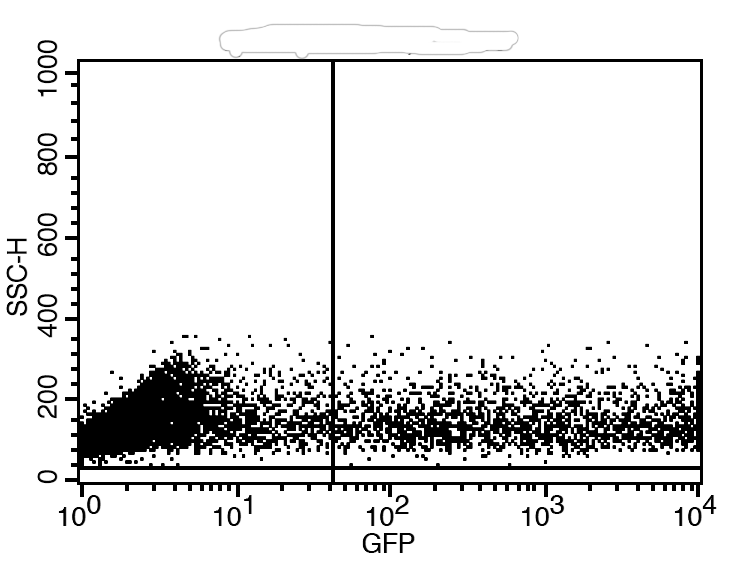

Supplement: Supplementary file 1 — S1: Transfection efficiency of novel cationic polyene lipids in another retinal cell line, D407. Comparison with Gene Juice, a commercial reagent, shows a similar trend. S2: Flow cytometry scatter plots for cell transfection with the novel cationic polyene lipids. [file 703253.f1.zip › EPC 6.tif]
